# Soluble Siglec-5 associates to PSGL-1 and displays anti-inflammatory activity

**DOI:** 10.1038/srep37953

**Published:** 2016-11-28

**Authors:** Marion Pepin, Soraya Mezouar, Julie Pegon, Vincent Muczynski, Frédéric Adam, Elsa P. Bianchini, Amine Bazaa, Valerie Proulle, Alain Rupin, Jerome Paysant, Laurence Panicot-Dubois, Olivier D. Christophe, Christophe Dubois, Peter J. Lenting, Cécile V. Denis

**Affiliations:** 1 Institut National de la Santé et de la Recherche Médicale, UMR_S 1176, Univ. Paris-Sud, Université Paris-Saclay, 94276 Le Kremlin-Bicêtre, France; 2 Aix Marseille Université, Inserm UMR_S 1076, (VRCM) Vascular Research Center of Marseille, 13385 Marseille, France; 3 Department of Biological Hematology, CHU Bicetre, Hopitaux Universitaires Paris Sud, AP-HP, Paris, France; 4 Institut de Recherche International Servier, Recherche Translationelle et Clinique Oncologie, 92150, Suresnes, France; 5 Institut de Recherches Servier, Unité de Recherche et de Découverte Cardiovasculaire, 92150, Suresnes, France

## Abstract

Interactions between endothelial selectins and the leukocyte counter-receptor PSGL1 mediates leukocyte recruitment to inflammation sites. PSGL1 is highly sialylated, making it a potential ligand for Siglec-5, a leukocyte-receptor that recognizes sialic acid structures. Binding assays using soluble Siglec-5 variants (sSiglec-5/C4BP and sSiglec-5/Fc) revealed a dose- and calcium-dependent binding to PSGL1. Pre-treatment of PSGL1 with sialidase reduced Siglec-5 binding by 79 ± 4%. In confocal immune-fluorescence assays, we observed that 50% of Peripheral Blood Mononuclear Cells (PBMCs) simultaneously express PSGL1 and Siglec-5. Duolink-proximity ligation analysis demonstrated that PSGL1 and Siglec-5 are in close proximity (<40 nm) in 31 ± 4% of PBMCs. *In vitro* perfusion assays revealed that leukocyte-rolling over E- and P-selectin was inhibited by sSiglec-5/Fc or sSiglec-5/C4BP, while adhesion onto VCAM1 was unaffected. When applied to healthy mice (0.8 mg/kg), sSiglec-5/C4BP significantly reduced the number of rolling leukocytes under basal conditions (10.9 ± 3.7 versus 23.5 ± 9.3 leukocytes/field/min for sSiglec-5/C4BP-treated and control mice, respectively; *p* = 0.0093). Moreover, leukocyte recruitment was inhibited over a 5-h observation period in an *in vivo* model of TNFalpha-induced inflammation following injection sSiglec-5/C4BP (0.8 mg/kg). Our data identify PSGL1 as a ligand for Siglec-5, and soluble Siglec-5 variants appear efficient in blocking PSGL1-mediated leukocyte rolling and the inflammatory response in general.

The inflammatory response involves a series of events that leads to the recruitment of circulating leukocytes to extravascular sites of inflammation^1^. Many molecular actors have been identified that contribute to this process, including the leukocyte-receptor P-selectin glycoprotein ligand-1 (PSGL1)^2^,^3^. This single-membrane receptor controls rolling of leukocytes on P- and E-selectin-expressing endothelial cells^4^,^5^. Indeed, PSGL1-deficiency is associated with delayed leukocyte recruitment in an experimental peritonitis model as well as with reduced leukocyte rolling *in vivo*^6^. The extracellular domain of PSGL1 is heavily glycosylated^5^ and the presence of sialic acids at the tip of the glycans is crucial to its interaction with P-selectin^2^.

The presence of the sialylated glycans on PSGL1 also makes this protein a potential ligand for a family of sialic-acid recognizing receptors: Siglecs or sialic acid-binding immunoglobulin-like lectins. This family consists of 14 different members, each of them with its own specificity for the various sialic acid structures and conformations^7^. Two different subfamilies may be distinguished: CD22-related and CD33-related Siglecs. The CD22-related subfamily is composed of 4 members, including the archetype of this family, Sialoadhesin or Siglec-1, and these Siglecs are relatively well conserved between species. The human CD33-related subfamily consists of 10 different members, which are poorly conserved between species^8^. Siglecs are selectively expressed in cells of haematopoietic origin, such as neutrophils, B cells and monocytes, with each Siglec having its own expression pattern^8^.

Among the Siglecs, Siglec-5 recognizes a remarkably wide spectrum of sialic acid structures, including α2-3, α2-6 and α2-8 linkage conformations, as well as the two most common mammalian sialic acid variants (N-acetylneuraminic acid and N-glycolylneuraminic acid)^9^. The protein structure of Siglec-5 distinguishes 4 extracellular immunoglobulin-like domains, a single transmembrane domain and a cytoplasmic tail^10^. It should be noted that different splice variants of Siglec-5 can be produced, including a soluble isoform^11^. In all variants, the N-terminal V-set domain contains the sialic acid binding domain^11^,^12^. The reported physiological role of Siglec-5 seems to be diverse and relates to cell-cell interactions, signaling, recognition of pathogens and endocytosis of ligands^7^,^10^,^13^,^14^,^15^,^16^,^17^.

Being intrigued by the notion that PSGL-1 and Siglec-5 are both expressed in many leukocytes, combined with the presence of sialic acid structures on PSGL1, we investigated the hypothesis that PSGL1 could serve as a ligand for Siglec-5. Our data suggest that PSGL1 and Siglec-5 co-localize at the surface of leukocytes, and that recombinant PSGL1 binds in a calcium-dependent manner to soluble Siglec-5. Furthermore, soluble Siglec-5 interferes with the rolling of leukocytes on P-selectin *in vitro*. Finally, soluble Siglec-5 reduces basal rolling of leukocytes *in vivo* and prevents the recruitment of leukocytes to sites of inflammation.

## Results

### Soluble Siglec-5 variants

**Figure 1:**
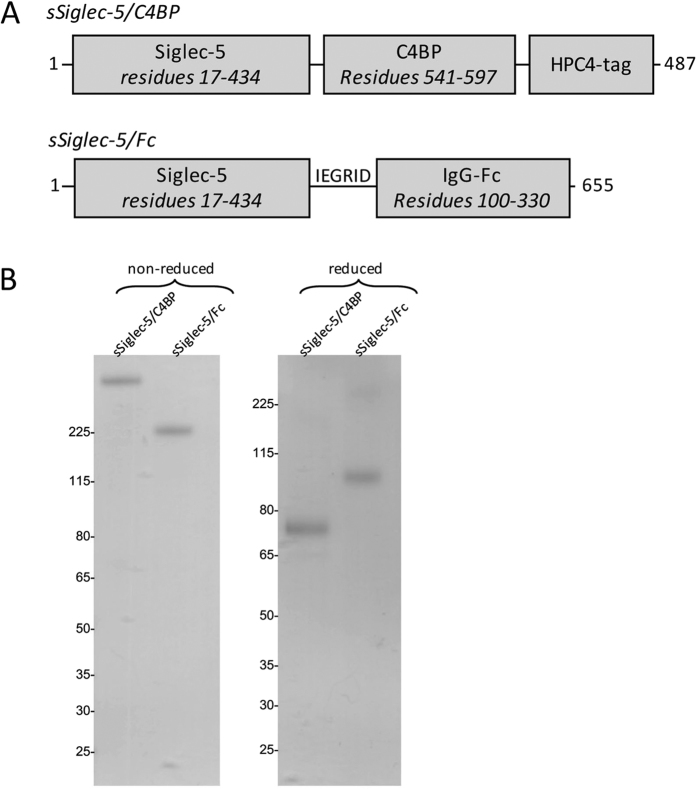
Soluble variants of Siglec-5. *Panel A*: Domain structures of sSiglec-5/C4BP and sSiglec-5/Fc which are used within this study. *Panel B*: Analysis of purified sSiglec-5/C4BP and sSiglec-5/Fc via SDS-Page and Coomassie-staining under non-reduced and reduced conditions.

To investigate whether Siglec-5 recognizes the ectodomain of PSGL1, two different Siglec-5 constructs of different cellular origin were used to explore their interaction with soluble PSGL1/Fc (sPSGL1/Fc): a commercially available dimeric soluble Siglec-5/Fc fusion protein (sSiglec-5/Fc) and a novel heptameric Siglec-5/C4BP fusion protein (sSiglec-5/C4BP; Fig. 1A). This latter protein consists of the Siglec-5 ectodomain fused to a 57-amino acid motif that mediates heptamerisation of the C4BP protein (residues 541–597)^18^. Indeed, purified sSiglec-5/C4BP migrates as a single-chain protein of approximately 525 kDa under non-reduced conditions. The apparent molecular weight of sSiglec-5/C4BP was estimated to be one-seventh of this value under reduced conditions (75 kDa; Fig. 1B), corresponding to the molecular weight of the 487-amino acid polypeptide, which harbors 8 sites for N-linked glycosylation.

### Interaction between the ectodomains of PSGL1 and Siglec-5

**Figure 2:**
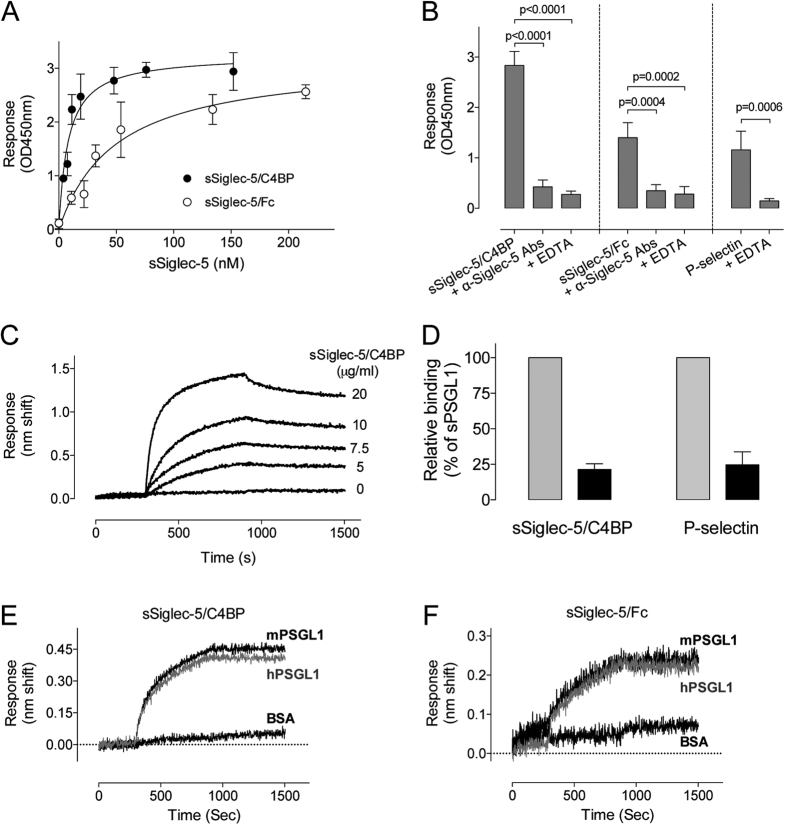
Interaction between ectodomains of Siglec-5 and PSGL1. *Panel A*: sSiglec-5/C4BP (closed circles) and sSiglec-5/Fc (open circles) were incubated with immobilized sPSGL1/Fc. Plotted is the response (OD450nm) *versus* sSiglec-5 concentration. Data represent mean ± SD of three independent experiments. *Panel B*: sSiglec-5/C4BP (10 μg/ml), sSiglec-5/Fc (10 μg/ml) or P-selectin (10 μg/ml) were pre-incubated with EDTA or polyclonal anti-Siglec-5 antibodies prior to the addition of sPSGL1/Fc-coated wells. Plotted is the response (OD450) for each of the preparations. P-values were determined in a 1-way ANOVA followed by a Tukey’s multiple comparison test. *Panel C*: Interaction between sPSGL1 and sSiglec-5/C4BP was examined using BLI-analysis. sPSGL1/Fc was immobilized onto amine-reactive biosensors and incubated with various concentrations of sSiglec-5/C4BP. A representative sensorgram of three independent experiments is plotted, showing the response (nm shift) versus time. *Panel D*: sPSGL1/Fc immobilized onto amine-reactive biosensors was incubated in the absence (grey bars) or presence (black bars) of sialidase (0.1 U/ml) before exposure to sSiglec-5/C4BP or P-selectin (both 10 μg/ml). Plotted is the residual response (expressed as percentage of response in the absence of sialidase, which was arbitrarily set at 100%) for both sSiglec-5/C4BP and P-selectin. *Panels E & F*: human or murine sPSGL1/Fc (hPSGL1 and mPSGL1) and BSA were immobilized onto amine-reactive biosensors and incubated with sSiglec-5/C4BP or sSiglec-5/Fc (7.5 μg/ml). Representative sensorgrams of two independent experiments is plotted, showing the response (nm shift) versus time.

Binding of Siglec-5 variants (0–215 nM) to immobilized sPSGL1/Fc (0.25 μg/well) was first tested in an immunosorbent assay, with bound Siglec-5 protein being probed using biotin-labeled polyclonal anti-Siglec-5 antibodies. Both sSiglec-5/C4BP and sSiglec-5/Fc displayed saturable and dose-dependent binding to immobilized sPSGL1/Fc (Fig. 2A). Half-maximal binding was calculated to be 8 ± 1 nM and 49 ± 11 nM for sSiglec-5/C4BP and sSiglec-5/Fc, respectively. Binding appeared to be specific, as the interaction of sSiglec-5/C4BP and sSiglec-5/Fc was inhibited in the presence of polyclonal anti-Siglec-5 antibodies as well as in the presence of EDTA (Fig. 2B). A similar EDTA-dependence was also observed for the binding of P-selectin to immobilized sPSGL1/Fc (Fig. 2B). Binding was further investigated via BLI-analysis using Octet-QK equipment. Again, sSiglec-5/C4BP displayed a dose-dependent binding (Fig. 2C). Specificity was tested by pre-incubating immobilized sPSGL-1/Fc with sialidase prior to incubation with sSiglec-5/C4BP. Sialidase-treatment of sPSGL1/Fc reduced binding of sSiglec-5/C4BP and P-selectin by 79 ± 4% and 75 ± 9%, respectively (mean ± SD; n = 3; Fig. 2D). Since *in vivo* experiments were included in this study, we further verified that sSigecl-5/C4BP and sSiglec-5/Fc can interact with murine PSGL1 as well. As depicted in Fig. 2E and F, both soluble Siglec-5 variants were similar in binding to human and murine sPSGL1/Fc. Taken together, our data indicate that the extracellular region of PSGL1 is able to associate with the extracellular region of Siglec-5 in a calcium-ion and sialic acid-dependent manner.

### PSGL1 and Siglec-5 are in close proximity at the leukocyte surface

**Figure 3:**
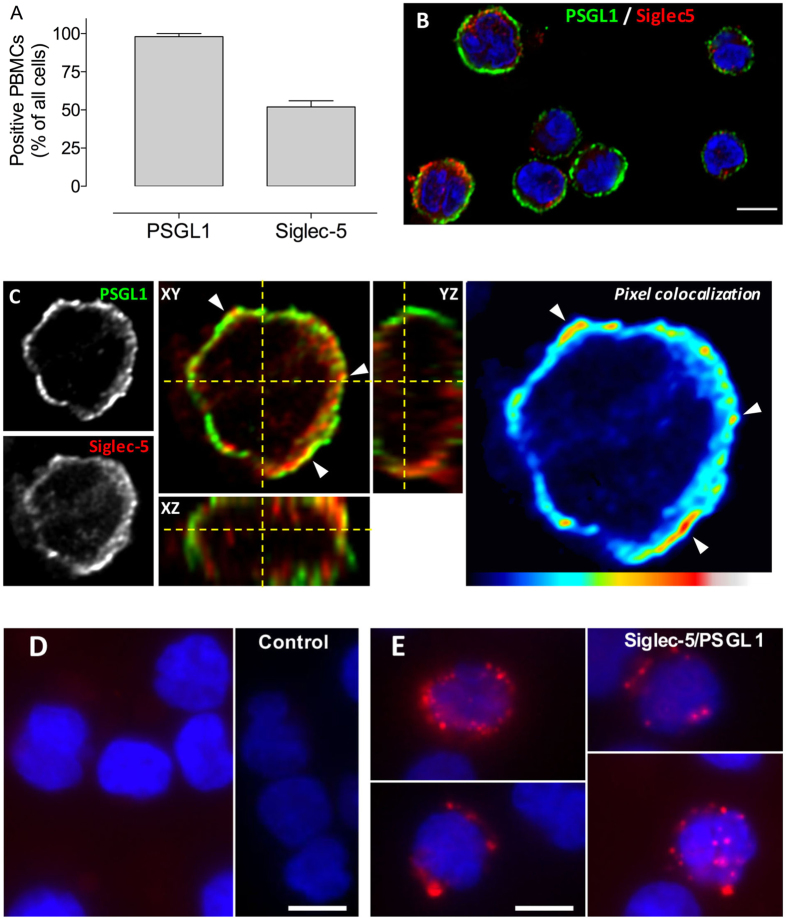
Immunostaining of PSGL1 and Siglec-5 on human PBMCs. *Panel A*: Purified human PBMCs were stained for PSGL1 and Siglec-5, and the number of positive cells was counted. Presented is the percentage positive cells of three independent experiments (mean ± SD; n = 30-60 cells *per* experiment). *Panel B*: Confocal microscopy image of a representative staining of PBMCs for PSGL1 (green) and Siglec-5 (red). Objective 40x. Bar represent 5 micron. *Panel C*: Confocal microscopy analysis of a representative cell co-expressing PSGL1 (green) and Siglec-5 (red). Cell stack was reconstituted in orthoview to visualize co-localization of two signals. Arrows indicate areas of colocalization. Z-depth is 0.5 μm, objective 63x. The pixel co-localisation heatmap was built using *Co-localization Threshold*-plugin Fiji Software. *Panels D*-*E*: Widefield microscopy images of a Duolink-PLA assay for PSGL1 and Siglec-5 incubated in the absence (*panel D*) or presence (*panel E*) of primary antibodies. Objective 40x. Bars represent 5 micron. For images in *panels B*-*D*-*E*, nuclei were counterstained with DAPI (blue).

We next explored the possibility that the naturally expressed proteins could also interact at the cell surface. When analyzing the expression of PSGL1 and Siglec-5 on freshly isolated PBMCs, we observed that 98 ± 2% (n = 3 PBMC isolations) of the cells stained positive for PSGL1 and 52 ± 4% for Siglec-5 (Fig. 3A-B), suggesting that about 50% of the cells express both PSGL1 and Siglec-5. We then analyzed co-localization between PSGL1 and Siglec-5 via double immunofluorescence staining. Signals for PSGL1 on Siglec-5-expressing cells indeed displayed some overlap with the signals for Siglec-5 (Fig. 3C). To confirm that both proteins truly associate at the cell surface, Duolink-PLA analysis was performed. This approach generates red fluorescent spots if proteins are located in a radius of 40 nm. No fluorescent spots were formed when either primary antibody was omitted from the assay (Fig. 3D). In contrast, distinct red spots were detected in about 30% of the cells when anti-PSGL1 and anti-Siglec-5 antibodies were used simultaneously (Fig. 3E). These data show that PSGL1 and Siglec-5 may interact also at the surface of PBMCs, indicating that binding is not restricted to the recombinant derivatives of these proteins.

### Soluble sSiglec-5/Fc reduces rolling of THP1-cells over E- and P-selectin *in vitro*

**Figure 4:**
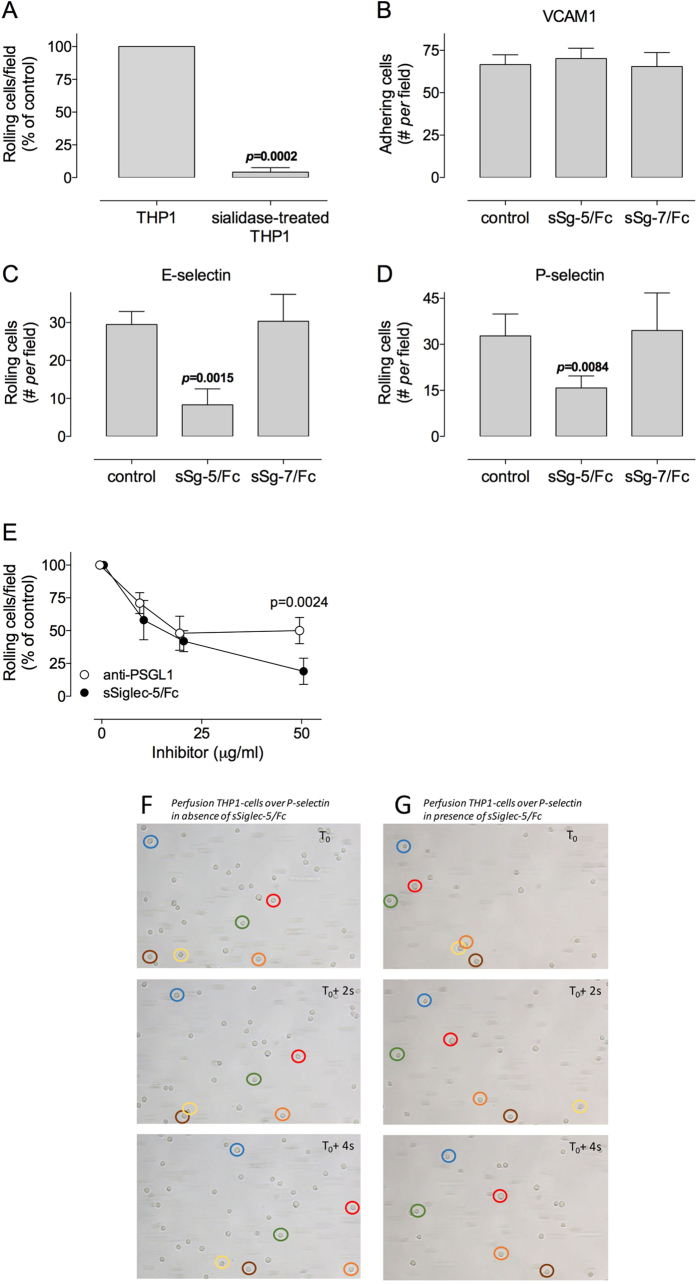
*In vitro* perfusion of THP1-cells. *Panel A*: THP1-cells were incubated with in the absence or presence of sialidase prior to perfusion over P-selectin-coated biochips. The number of rolling cells/microscopic field were determined and arbitrarily set at 100% for non-desialylated THP1-cells. P-value was determined in a two-tailed unpaired t-test. *Panel B*: THP1-cells were incubated in the absence or presence of soluble Siglec-5/Fc (sSg-5/Fc) or Siglec-7/Fc (sSg-7/Fc) prior to perfusion over VCAM1-coated biochips. Plotted is the number of adhering cells/microscopic field. *Panels C & D*: THP1-cells were incubated in the absence or presence of soluble Siglec-5/Fc (sSg-5/Fc; 10 μg/ml) or Siglec-7/Fc (sSg-7/Fc; 10 μg/ml) prior to perfusion over E-selectin or P-selectin-coated biochips. Plotted is the number of rolling cells/microscopic field. P-values were determined in a 1-way ANOVA followed by Dunnett’s test. *Panel D*: THP1-cells were incubated in the absence or presence of sSiglec-5/Fc or anti-PSGL1 antibodies prior to perfusion over P-selectin-coated biochips. Plotted is the number of rolling cells/microscopic field, with this number being set as 100% in the absence of sSiglec-5/Fc or anti-PSGL1 antibodies. P-value was calculated via multiple t-tests according to the Holm-Sidak method. *Panels F & G*: representative image-sequence over a 4-sec period of THP1-cells rolling over P-selectin in the absence (panel F) or presence (panel G) of sSiglec-5/Fc. For each image-sequence, six cells have been highlighted to visualize their movements over time. For panels A–E: data represent mean ± SD of 3–5 perfusions. For each perfusion, a minimum of 3 fields were analyzed. All perfusions were performed at 0.5 dyn/cm^2^.

Considering that sSiglec-5 variants are able to interact with PSGL1, we explored the possibility that sSiglec-5 could be used to interfere with PSGL1 mediated leukocyte rolling. First, we compared rolling of THP1-cells that were pre-incubated in the absence or presence of sialidase (0.1 U/ml). The number of THP1-cells rolling over immobilized P-selectin was reduced by >98% for sialidase-treated THP1-cells compared to non-treated cells (Fig. 4A), confirming that P-selectin mediated rolling requires the presence of sialic acids. In a next series of experiments, the effect of a single concentration of dimeric sSiglec-5/Fc on the adhesion and/or rolling of THP1-cells on various receptors was tested using a microfluidic perfusion system (shear stress: 0.5 dyn/cm^2^). As a control, sSiglec-7/Fc was used, which recognizes a different array of sialic acid conformations compared to sSiglec-5/Fc^9^. In the presence of sSiglec-5/Fc or sSiglec-7/Fc (10 μg/ml), the number of cells adhering to VCAM1 was unaffected (Fig. 4B). In contrast, rolling on E-selectin or P-selectin-coated coverslips was reduced by 72 ± 14% (n = 3; p = 0.0015) and 52 ± 12% (n = 5; p = 0.0084), respectively, in the presence of sSiglec-5/Fc, but not in the presence of sSiglec-7/Fc (Fig. 4C & D). We subsequently compared sSiglec-5/Fc to anti-PSGL1 antibodies. Both the anti-PSGL1-antibodies and sSiglec-5/Fc reduced rolling of THP1-cells over P-selectin in a dose-dependent manner (Fig. 4E). Moreover, both anti-PSGL1-antibodies and sSiglec-5/Fc were equally efficient in reducing rolling of THP1-cells at concentrations of 10 and 20 μg/ml. However, no further decrease in rolling was observed at 50 μg/ml anti-PSGL1-antibodies (50 ± 10% maximal inhibition), whereas rolling was reduced by 81 ± 10% in the presence of 50 μg/ml sSiglec-5/Fc (p = 0.0024 compared to anti-PSGL1 antibodies). Examples of image-sequences depicting rolling of THP1-cells over P-selectin in the absence or presence of sSiglec-5/Fc are shown in Fig. 4F and G. Our data suggest that sSiglec-5/Fc is able to specifically interfere with rolling of leukocytes over P-selectin.

### Soluble sSiglec-5 variants reduce rolling of PBMCs and PMNs over P-selectin *in vitro*

**Figure 5:**
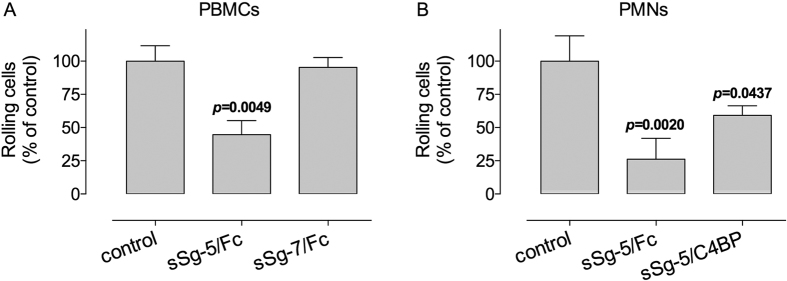
*In vitro* perfusion of primary leukocytes. *Panel A*: Freshly isolated PBMCs were incubated with in the absence or presence of soluble Siglec-5/Fc (sSg-5/Fc; 10 μg/ml) or Siglec-7/Fc (sSg-7/Fc; 10 μg/ml) prior to perfusion over P-selectin-coated biochips. Perfusion was performed at 2 dyn/cm^2^. *Panel B*: Freshly isolated PMNs were incubated with in the absence or presence of soluble Siglec-5/Fc (sSg-5/Fc; 10 μg/ml) or sSiglec-5/C4BP (sSg-5/C4BP; 10 μg/ml) prior to perfusion over P-selectin-coated biochips. Perfusion was performed at 0.5 dyn/cm^2^. For both panels, the number of rolling cells/microscopic field were determined and arbitrarily set at 100% for the control in the absence of Siglecs. Data represent mean ± SD of 3–4 perfusions. For each perfusion, a minimum of 3 fields were analyzed. P-values were determined in a 1-way ANOVA followed by Dunnett’s test.

To investigate if soluble Siglec-5 variants also modify rolling of primary leukocytes over P-selectin, perfusion experiments using freshly isolated PBMCs and PMNs were performed. Rolling of PBMCs over P-selectin was reduced by 55 ± 11% (p = 0.0049; n = 3) in the presence of sSiglec-5/Fc, while unaffected by the presence of sSiglec-7/Fc (Fig. 5A). As for PMNs, both sSiglec-5/Fc and sSiglec-5/C4BP significantly reduced rolling of these cells over P-selectin. Rolling was reduced by 26 ± 16% (p = 0.0020; n = 4) and 41 ± 7% (p = 0.0437; n = 5), respectively (Fig. 5B). Thus, sSiglec-5 also interferes with the rolling of PBMCs and PMNs over P-selectin.

### sSiglec-5/C4BP reduces basal leukocyte rolling *in vivo*

**Figure 6:**
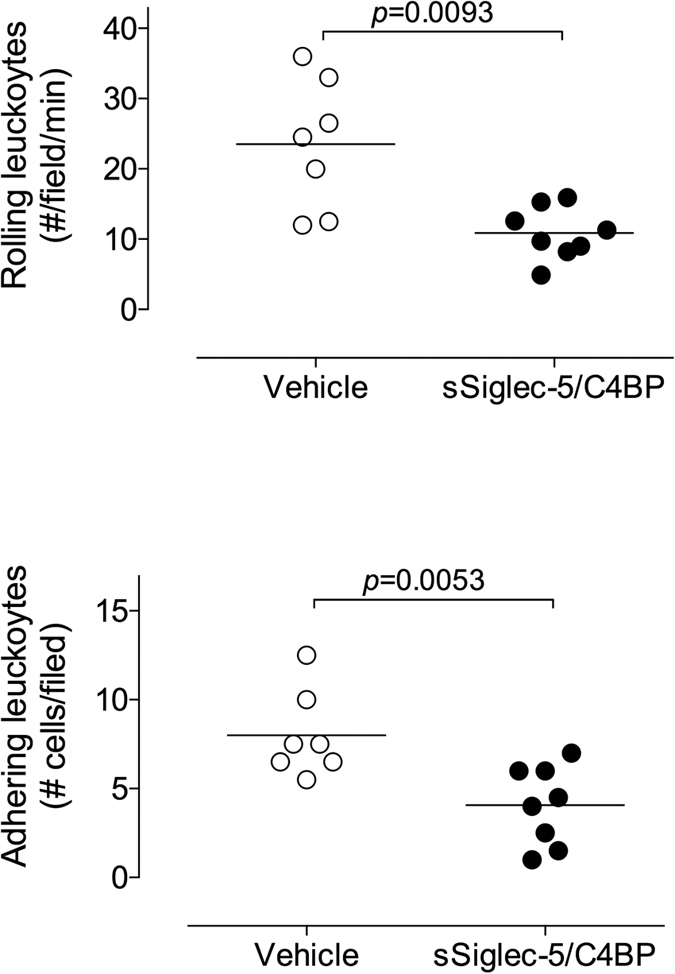
Basal leukocyte rolling *in vivo*. Leukocyte rolling in mesenteric venules (25–35 micron) was recorded via video-microscopy for 10 min. Videos were then analyzed for the number of rolling leukocytes (*panel A*) and the number of adhering leukocytes (defined as leukocytes adherent for at least 30 sec; *panel B*). *Panel A*: Each data point represents the average number of rolling cells/field/min for each mouse. For each mouse, 2–3 vessel segments were analyzed. *Panel B*: Each point represents the cumulative number of adherent cells per field over the 10 min observation period. For each mouse, 2 vessel segments were analyzed. Mice received either vehicle (open circles) or sSiglec-5/C4BP (0.8 mg/kg via injection in the lateral tail vein; closed circles). P-values were determined via a Mann-Whitney test.

To investigate whether sSiglec-5 affects leukocyte rolling *in vivo*, we first studied the effect of infusion of sSiglec-5/C4BP infusion on basal leukocyte rolling in mice. To this end, mice (13–18 g; 3–5 weeks of age) received a single bolus of vehicle or sSiglec-5/C4BP (0.8 mg/kg). After preparation of the mesenteric venules (25–35 μm), leukocyte rolling was monitored for 10 min in two separate vessels in 8–9 separate mice. The number of rolling leukocytes under basal conditions was significantly lower in sSiglec-5/C4BP-treated mice compared to vehicle-treated mice (10.9 ± 3.7 versus 23.5 ± 9.3 rolling leukocytes/field/min, respectively; *p* = 0.0093; Fig. 6A). Moreover, the number of adhering leukocytes (defined as adhering for >30s) was also reduced significantly in Siglec-5/C4BP-treated mice compared to vehicle treated mice (4.1 ± 2.2 versus 8.0 ± 2.4 adhering leukocytes/field, respectively; *p* = 0.0053; Fig. 6B). These data suggest that sSiglec-5/C4BP has the potential to modulate leukocyte rolling and subsequent adhesion *in vivo*.

### sSiglec-5/C4BP prevents leukocyte recruitment in a TNFα-induced model of sterile inflammation

**Figure 7:**
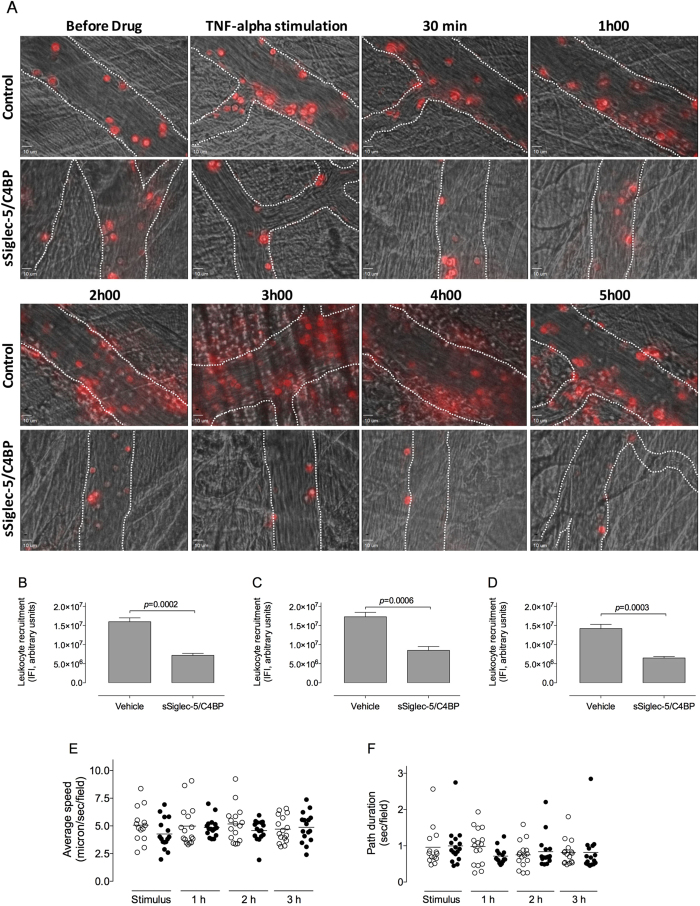
Involvement of sSiglec-5/C4BP in the kinetics of leukocyte recruitment during a sterile inflammation in mice. Leukocytes were labeled with fluorescent dye Syto59. *Panel A*: Representative images depicting the course of leukocyte recruitment during a sterile inflammation over time in control mice or mice treated with sSiglec-5/C4BP (0.8 mg/kg via injection in the lateral tail vein). *Panels B*-*C*-*D*: The total Integral Fluorescent Intensity (IFI) over the 5-h observation period was quantified in control mice or in mice treated with Siglec-5. Data represent the IFI of the full microscopic field (panel B), the IFI of the vessels (panel C) or the IFI of the surrounding tissue (panel D). Plotted are mean ± SEM from 4 mice *per* group and 4 vessels *per* mouse (n = 16). P-values were determined using a two-tailed unpaired t-test. *Panels E & F*: the average rolling velocity (panel E) and path duration (panel F) were determined for each vessel segment of each mouse included in the study at 4 different time-points during the observation period. Each data point represents the mean of 3–4 cells.

We next addressed the question whether sSiglec-5/C4BP could modulate leukocyte recruitment in a sterile model of inflammation. To this end, mice were given vehicle or a single bolus of sSiglec-5/C4BP (0.8 mg/kg). Subsequently, the cremaster of male mice was prepared and a TNFα-releasing gel was placed on the cremaster muscle. Recruitment, rolling and adhesion of Syto59-labeled leukocytes was monitored over a 5-h period using intravital spinning-disk real-time confocal microscopy of the cremaster vasculature. This approach allowed the separate quantification of leukocyte recruitment in the vessels and in the surrounding tissues. In vehicle-treated mice, placement of the TNFα-gel resulted in a near immediate increase in leukocyte accumulation in the vessel wall, which lasted over a prolonged period of time (Fig. 7A). In addition, a marked infiltration of leukocytes into the tissue was observed starting 2 h after TNFα-stimulation, while fewer leukocytes extravasated upon sSiglec-5/C4BP treatment (Fig. 7A). We then quantified the fluorescent signal that was produced under both conditions over the 5-h period (integrated fluorescence intensity: IFI for 4 mice/condition and 4 vessels segments/mouse analyzed), and three values were compared: the IFI of the full microscopic field (Fig. 7B), the IFI of the vessels (Fig. 7C) or the IFI of the surrounding tissue (Fig. 7D). For each of these analyses, the total IFS over the 5-h period was significantly reduced for mice treated with sSiglec-5/C4BP compared to vehicle treated mice. These data point to a powerful anti-inflammatory potential of sSiglec-5/C4BP.

## Discussion

Siglecs are widely expressed in immune cells of hematopoietic origin, and it seems conceivable that they participate in the immune response. Siglec-5 is a well-characterized member of this family, and its expression pattern among leukocytes has been reported in detail. Flow cytometric analysis revealed that Siglec-5 is present on neutrophils, monocytes, and B-cells, but not on T-cells^10^,^19^. This study focused on the potential association between Siglec-5 and PSGL1. The latter protein is crucial for the initial recruitment of immune cells to the endothelial cells that line the vasculature, and its function relies on the presence of sialic acids covering its glycan structures^2^. The presence of these sialic acids makes PSGL1 a potential ligand for Siglecs. In the present study, we show that recombinant variants of PSGL1 and Siglec-5 interact in a calcium-ion and sialic acid-dependent manner. This interaction also occurs at the surface of circulating leukocytes that co-express PSGL1 and Siglec-5, given the co-localization we observed when analyzing PBMCs. Finally, soluble Siglec-5 interferes with interactions between PSGL1 and P-selectin *in vitro*, and displays strong anti-inflammatory activity *in vivo.*

When analyzing PBMCs, we observed that about 50% of the cells express both Siglec-5 and PSGL1. This 50% corresponds to the potential Siglec-5-positive fraction (monocytes and B-cells) that is present in Ficoll-purified PBMCs^19^,^20^. Most likely, the Siglec-5 negative population corresponds to T-cells. Two distinct approaches were used to determine co-localization of both proteins at the cellular surface: classic immune-staining (Fig. 3C) and Duolink-PLA analysis (Fig. 3E). This latter approach generates red fluorescent spots if proteins are located within a range of 40 nm, a range compatible with a true co-localization of proteins.

In another set of experiments, we have tried to confirm the interaction between Siglec-5 and PSGL1 at the cell-surface via co-immunoprecipitation experiments. However, numerous attempts remained unsuccessful, and control experiments using purified proteins revealed that the denaturating conditions needed to free PSGL1 and/or Siglec-5 from the cellular membrane resulted in dissociation of the PSGL1/Siglec-5 complex. The technical limitations therefore prevented us to confirm the interaction of both proteins via this experimental approach.

Despite the technical limitations of the immuno-precipitation, the notion that the ectodomains of PSGL1 and Siglec-5 interact was clearly demonstrated when using recombinant purified proteins. These experiments readily revealed a dose-dependent, saturable and specific interaction between both proteins (Fig. 2). Control experiments using inhibitory antibodies, EDTA and de-sialylated sPSGL1/Fc confirmed the specificity of the interaction (Fig. 2). The observation that de-sialylated sPSGL1/Fc displayed a strongly reduced binding to sSiglec-5/C4BP indicates that the interaction relies to a large extent on the sialic acids present on sPSGL1/Fc. This is in agreement with the majority of the ligands described for Siglec-5 in particular and the Siglec-family in general. However, it is important to mention that not all Siglec-5 ligands are bound in a sialic acid-dependent manner. For instance, Group B-streptococcus-protein and coagulation factor VIII have both been reported to interact with Siglec-5 without the need for sialic acids^16^,^21^. Our data do not exclude the possibility that the interaction between PSGL1 and Siglec-5 is of a mixed nature, involving both sialic acid-dependent and -independent types of interactions.

In the present study, we have not explored the physiological consequences of PSGL1 and Siglec-5 interacting at the cell-surface, as this will be part of a separate study. However, it is tempting to speculate on two issues in this regard. First, Siglecs, including Siglec-5, are generally known to associate with sialic acid-containing receptors at the cell-surface of under resting conditions, while unmasking these receptors upon cell stimulation^22^. It is possible that part of the PSGL1 molecules is associated with Siglec-5 under resting conditions, thereby masking the sialic acids of the PSGL1 glycans. Potentially this may prevent excessive premature leukocyte rolling. Upon leukocyte stimulation, Siglec-5 would then release PSGL1 to enhance PSGL1-mediated rolling. Second, it has been reported that leukocytes may produce soluble Siglec-5 variants^11^. Further, Biedermann and colleagues have found that plasma contains about 75 ng/ml of soluble Siglec-5, and amount that can increased 3–4 fold under pathological conditions^23^. It can not be excluded that increased levels of soluble Siglec-5 may modulate leukocyte rolling under these conditions.

Instead of addressing the physiological consequences, we directed our investigations to explore the potential of soluble Siglec-5 variants to interfere with PSGL1-dependent leukocyte rolling. In preliminary experiments, we used a monomeric soluble Siglec-5 variant, which we have described previously^16^. Although interacting with PSGL1 (data not shown), it did so rather inefficiently. Indeed, glycan-protein interactions are often of relatively low affinity and increasing valency via clustering often improves the interaction efficiency^24^,^25^,^26^. To overcome this limitation, two variants were used in this study: a dimeric sSiglec-5/Fc variant and a heptameric sSiglec-5/C4BP variant. The heptameric variant was designed based on a report by Libyh and coworkers, who described a scFv anti-Rh(D) antibody fused to the 57-amino acid peptide motif of C4BP that mediates heptamerization of the protein^18^. Indeed, upon expression in HEK293-cells, the sSiglec-5/C4BP was efficiently secreted as a single multivalent protein (Fig. 1). For both sSiglec-5/Fc and sSiglec-5/C4BP, we were able to detect efficient binding to PSGL1 *in vitro* (Fig. 2).

*In vitro* perfusion studies using human THP1-cells revealed that adhesion to VCAM1 was unaffected by sSiglec-5/Fc at a dose of 20 μg/ml (Fig. 4). The notion that THP1-cells were able to adhere to VCAM1 was perhaps surprising. However, the shear rates used in these particular experiments were relatively low (0.5 dyn/cm^2^). Moreover, resting THP1-cells express the VCAM1-ligand α4/β1, and previous studies have shown that part of these α4/β1 molecules is in its activated conformation^27^. We anticipate that the presence of α4/β1 in its active conformation in combination with the low shear is compatible with the direct adhesion of the THP1-cells to VCAM1.

In contrast to the adhesion to VCAM1, rolling on E- and P-selectin was reduced by about 2-fold in the presence of a similar dose of sSiglec-5/Fc (Fig. 4). This effect seemed specific for Siglec-5, as another member of the Siglec-family, Siglec-7, was unable to reduce rolling of THP1-cells on both selectins. Furthermore, sSiglec-5/Fc-mediated inhibition was dose-dependent and to a large extent similar to that of anti-PSGL1 antibodies (Fig. 4). However, at higher doses (50 μg/ml), sSiglec-5/Fc was more efficient than anti-PSGL1 antibodies in reducing THP1-cell rolling. One possibility is that at these concentrations, sSiglec-5/Fc also interferes with the adhesion of another P-selectin ligand. A potential candidate is ESL-1, which is expressed on THP1-cells and it binds to P-selectin in a glycan-dependent manner^28^,^29^. The specificity of sSiglec-5/Fc was not limited to THP1-cells, but this soluble protein also reduced rolling of primary leukocytes, both PBMCs and PMNs (Fig. 5). Furthermore, not only sSiglec-5/Fc but also its heptameric variant sSiglec-5/C4BP reduced rolling of PMNs *in vitro* (Fig. 5). Of note, a shear stress of 0.5 dyn/cm^2^ was used in most of the perfusion assays. Although relatively weak compared to the shear stress present in post-capillary venules (minimum 2.8 dyn/cm^2^, with an average of 15 dyn/cm^2^), 0.5 dyn/cm^2^ has been used in other studies concerning the rolling of leukocytes over P-selectin as well^30^,^31^. Lawrence and coworkers have reported that 0.5 dyn/cm^2^ represents the actual lower threshold to enhance rolling on P-selectin^31^. Nevertheless, to ensure that sSiglec-5 is also effective at higher shear stress, perfusion experiments using PMBCs were performed at 2 dyn/cm^2^ and also under these conditions rolling was reduced in the presence of sSiglec-5/Fc (Fig. 5A). Thus, it seems likely that sSiglec-5/Fc is indeed able to interfere with P-selectin/PSGL1 interactions.

To test its *in vivo* potential to modulate leukocyte behavior, we initially examined leukocyte rolling under basal conditions using a single dose of sSiglec-5/C4BP. In each of the 8 mice that received sSiglec-5/C4BP, the number of rolling leukocytes was significantly reduced compared to control mice (Fig. 6). We considered the possibility that the effect of sSiglec-5/C4BP would fade out during the examination period. However, similar differences between sSiglec-5/C4BP-receiving and control mice were observed in vessels examined at the start and at the end of a session, which routinely be separated by 20–40 minutes. It should be noted that the reduced rolling is less pronounced than observed in the *in vitro* experiments. This could be due to to sSiglec-5/C4BP interacting with other sialylated proteins present in the vascular system, and higher doses of sSiglec-5/C4BP could be used to bypass this restriction. We also considered the option to use a murine variant of Siglec-5 to this end, as it may provide higher specificity for murine ligands. However, human Siglec-5 is not well conserved between species, and no appropriate murine counterpart is available^32^. Interestingly, we also observed that sSiglec-5/C4BP reduced the number of adherent cells (Fig. 6B). PSGL1-dependent rolling over P-selectin is known to induce the Syk-mediated signaling pathway, resulting in activation of LFA-1^33^. This allows LFA-1 to interact with its counter-receptor ICAM-1, thereby facilitating firm adhesion of the leukocytes. By blocking PSGL1/P-selectin interactions, sSiglec-5 may prevent the activation of LFA-1, and consequently reduce firm adhesion.

The therapeutic potential of sSiglec-5/C4BP was examined in a sterile model of inflammation, in which the inflammatory response is triggered by contact with a TNFα-containing gel. Real-time video monitoring over a 5-h period allowed us to visualize and quantify the full process of leukocyte recruitment and extravasation. The exteriorization of cremaster muscle for 5 h may potentially induce inflammation. However, similar to models described by others^34^,^35^,^36^, the cremaster muscle is superfused with thermocontrolled bicarbonate-buffer solution, which efficiently suppresses the development of a spontaneous inflammatory response. Indeed, while validating this model, no inflammation was observed when the cremaster muscle was exposed to a control gel (agarose/PBS) during at least 5 h. Therefore, the inflammatory response observed is selectively due to the presence of TNFα in the gel. A second potential point of concern is that blood flow may decrease during the 5-h anesthesia. Although not quantified using a blood flow-probe, we could observe blood flow in real-time in the intra-vital setting. We did not observe marked differences in blood flow over time in the mice that were included in the data analysis. We further chose to use the TNFα/agarose gel rather than to inject TNFα intra-scrotum (i.s.). The main reason to do so, is that following i.s. injection, it takes approximately 4 h before the effects of TNFα become visible^37^,^38^. By applying the TNFα/agarose gel, also the early steps in the inflammatory response can be monitored^34^.

**Figure 8:**
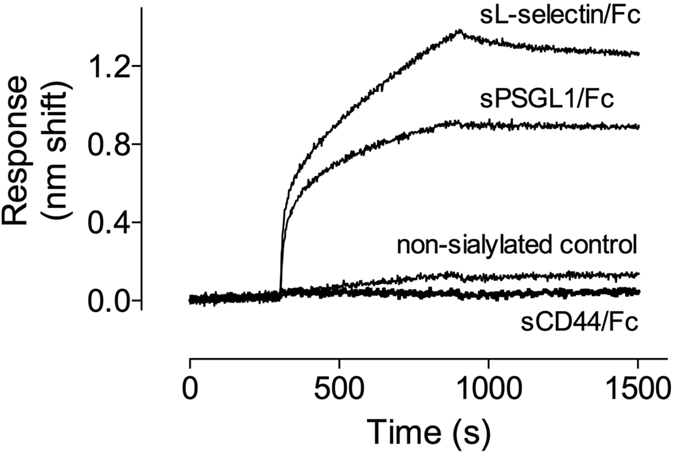
Interaction between sSIglec-5/C4BP and different adhesion proteins. Interaction between sSiglec-5/C4BP and sPSGL1/Fc, sL-selectin/Fc and sCD44/Fc was examined using BLI-analysis. sSiglec-5/Fc (10 μg/ml) was immobilized onto amine-reactive biosensors and incubated with a single concentration (10 μg/ml) of sPSGL1/Fc, sL-selectin/Fc and sCD44/Fc. A non-sialylated bacterial protein was used as negative control. A representative sensorgram (of three independent measurements) is plotted, showing the response (nm shift) versus time.

As compared to vehicle-treated mice, the recruitment of leukocytes was strongly reduced in mice that received sSiglec-5/C4BP (Fig. 7), confirming that this protein may interfere with the interaction between PSGL1 and selectins. It was surprising, however, that the effect of sSiglec-5/C4BP persisted over the full 5-h observation period. Indeed, deficiency of PSGL1 in mice is associated with a reduced leukocyte recruitment at the early stage (2 h) of the inflammatory process, but was only minimally abnormal at the later timepoints^6^. In contrast, mice that have a double deficiency of P- and E-selectin have a severe impairment of leukocyte recruitment over a prolonged period of time^39^,^40^. The results we obtain with sSiglec-5/C4BP mimic to some extent the combined deficiency of P- and E-selectin, with a defect over a prolonged timescale. This suggests that the effects of sSiglec-5/C4BP are not limited to P-selectin/PSGL1 interactions, but have a wider impact. This seems compatible with the observation that sSiglec-5/Fc also interfered with the rolling of THP1-cells over E-selectin (Fig. 4C). Moreover, sSiglec-5/Fc reduced leukocyte rolling on P-selectin to a larger extent compared to anti-PSGL1 antibodies (Fig. 4E). Apparently, Siglec-5 is able to interact with a wider range of sialic acid-containing proteins involved in the inflammatory response. One possible option in this regard is that sSiglec-5/C4BP interferes with the adhesion mediated by the L-selectin axis, given that sL-selectin/Fc displayed efficient binding to sSiglec-5/C4BP in preliminary experiments (Fig. 8). L-selectin is a sialylated protein, and human L-selectin has been described as a ligand for E-selectin but not P-selectin^41^. Interestingly, de-sialysation of L-selectin coincides with a loss of binding to E-selectin^41^. It is possible therefore that sSiglec-5 binding may interfere with the L-selectin/E-selectin interaction. In parallel, sSiglec-5 binding to PSGL1 may prevent the association between PSGL1 and L-selectin as well. In contrast, no interaction between sSiglec-5/C4BP and the E-selectin ligand CD44 could be detected.

Irrespective of its precise mode of function, our *in vivo* data suggest a potent anti-inflammatory action by sSiglec-5/C4BP. In recent years, many pathological processes have been described to be dependent on the PSGL1/P-selectin pathway, including atherosclerosis^42^, venous thrombosis^43^, acute lung injury^44^, tumor metastasis^45^, and viral infections^46^,^47^. It would therefore be of interest to further explore the potential beneficial effects of sSiglec-5/C4BP in other pathological conditions.

## Material and Methods

### Ethics statement

#### Mice

Housing and experiments of mice were done in accordance with French regulations and the experimental guidelines of the European Community. Animal experiments were approved by the local ethical committees of Paris-Sud (Comité d’éthique en experimentation animale n° 26) and Aix-Marseille University (Comité d’éthique en experimentation animale n° 14) under numbers 201507271259485 and 43-17102012, respectively.

#### Human subjects

Blood was obtained from healthy volunteers via the local blood donor service of the University Hospital Bicetre (CHU-Bicetre), approval of which was provided by the Institutional Review Board of CHU-Bicetre. All participants gave informed consent for the sampling of blood for scientific purposes *per* the Declaration of Helsinki. Experiments were performed in accordance with the relevant guidelines and regulations.

### Proteins

Recombinant soluble Siglec-5 fused to human Fc (sSiglec-5/Fc; cat# 1072-SL, produced in mouse myeloma cell-line NS0), human and murine soluble recombinant PSGL1 fused to human Fc (sPSGL1/Fc; cat# 3345-PS & cat# 7407-PS), recombinant P-selectin (P-selectin; cat# ADP3), VCAM1 (cat# ADP5), soluble recombinant human L-selectin fused to human Fc (sL-selectin/Fc; cat# 728-LS), soluble recombinant human CD44 fused to human F (sCD44/Fc; cat# 3660-CD), polyclonal goat-anti Siglec-5 antibodies (cat# AF1072 & BAF1072), and monoclonal mouse anti-Siglec 5 antibody (cat# MAB10721) were all obtained from R&D Systems (Lille, France). Monoclonal mouse anti-PSGL1-antibody (cat# 556052) was from BD Pharmigen (Le Pont de Claix, France). Monoclonal mouse anti-P-selectin-antibody (cat# 14-0628) was from eBioscience (Paris, France). Peroxidase-labeled polyclonal goat-anti-mouse IgG (cat# P0447) was from Dako (Les Ulis, France). Cy3-labeled donkey anti-goat-antibodies (cat# ab6949) were from Abcam (Paris, France). AlexaFluor488-labeled donkey anti-mouse-antibodies (cat# A-21202) were from ThermoFisher Scientific (Illkirch, France). A fusion protein consisting of the Siglec-5 ectodomain (residues 17–434), residues 541–597 from C4b-binding protein (C4BP) and the epitope recognized by antibody HPC4 (designated sSiglec-5/C4BP; Fig. 1) was produced in HEK293-cells and purified via HPC4-mediated immune-affinity chromatography similarly as described^16^. Both purified sSiglec-5/C4BP and sSiglec-5/Fc were homogeneous as assessed via SDS-PAGE and Coomassie-staining (Fig. 1; see also Supplementary Fig. S1). Bovine serum albumin (BSA) and sialidase were from Sigma Aldrich (Saint-Quentin Fallavier, France).

### Peripheral mononuclear and polymorphonuclear cells

Peripheral Blood Mononuclear Cells (PBMCs) and polymorphonuclear cells (PMNs) were freshly isolated from blood obtained from healthy volunteers by Ficoll-Plaque density centrifugation. Eventual contaminating erythrocytes were removed by incubation with ice-cold lysis buffer (0.155 mM NH_4_Cl, 7.4 mM KHCO_3_, 0.1 mM EDTA, pH7.4). Cells were resuspended in Hank’s buffered salt solution (Gibco Life Technologies, Saint Aubin, France).

### Immunostaining

Purified PBMCs were fixed in solution with paraformaldehyde (4%) and dried on glass coverslips. Cells were re-hydrated with phosphate-buffered saline (PBS) and further permeabilized via incubation with PBS supplemented with 0.05% Tween-20 (PBS-T) and Triton X-100 (0.1%) for 10 min at room temperature. Coverslips were blocked with PBS-T containing 5% BSA for 30 min at room temperature. Cells were incubated with primary antibodies (polyclonal anti-Siglec-5 and monoclonal anti-PSGL1 antibodies). Bound antibodies were probed with Cy3-labeled donkey anti-goat antibodies and AlexaFluor488-labeled donkey anti-mouse antibodies, respectively. Nuclei were counterstained with 1 μg/ml of 4′,6′-diamino-2-phenylindole (DAPI) and coverslips were mounted in ProlongGold antifade reagent (ThermoFisher Scientific).

For Duolink experiments to detect close proximity between PSGL1 and Siglec-5, a similar double immunostaining was performed with the secondary antibodies replaced by secondary PLA-probes from the Duolink kit (Sigma Aldrich). The remainder of the protocol was conducted according to the manufacturer’s recommendations and the orange (555 nm wavelength) detection kit was used. Hybridization between the two PLA plus and minus probes leading to fluorescent signal only occurs when the distance between the two antigens is less than 40 nm.

### Microscopic analysis

Widefield microscopy images were acquired on an AxioImager A1 (Carl Zeiss, Oberkocher, Germany) and an Eclipse E600 (Nikon, Japan). Confocal microscopy analysis images were acquired on a LSM700 (Carl Zeiss) and cell stacks were further deconvolved using Huygens software (Scientific Volume Imaging, Hilversum, The Netherlands). Images were analyzed using ImageJ software.

### Immunosorbent assays

sPSGL1/Fc (0.25μg/well) was adsorbed to microtiter wells in 50 mM NaCO_3_, 10 mM NaHCO_3_ (pH9.6) overnight at 4 °C. Unoccupied sites were blocked in Tris-buffered saline with 2.5 mM CaCl_2_, 0.1% Tween-20, and 3% BSA for 2 h at 37 °C. Wells were incubated with various concentrations of sSiglec-5/C4BP or sSiglec-5/Fc (0–4 μM). Bound sSiglec-5 was probed using biotin-labeled polyclonal goat anti-Siglec-5 antibodies and peroxidase-labeled streptavidin and detected via hydrolysis of 3,3′,5,5′-tetramethylbenzidine (TMB, Thermofisher Scientific). Optical density (OD) was measured at 450 nm, and OD-values were corrected for OD-values obtained for the binding of sSiglec-5 to non-coated wells, which represented <10% of the OD-values. In control experiments, immobilized sPSGL1/Fc was incubated with sSiglec-5 (0.6 μM) in the presence of polyclonal anti-Siglec-5 antibodies (6 μM) or EDTA (5 mM). In other experiments, immobilized sPSGL1/Fc was incubated with P-selectin in the absence or presence of EDTA (5 mM). Bound P-selectin was probed using a monoclonal mouse anti-P-selectin antibody and detected using peroxidase-labeled polyclonal goat-anti mouse IgG antibodies.

### Biolayer interferometry analysis

In complementary experiments, binding of sPSGL1/Fc to immobilized sSiglec-5/C4BP was determined via biolayer-interferometry (BLI)-analysis using Octet-QK equipment. First, sPSGL1/Fc (10 μg/ml) was immobilized onto amine-coupling biosensors and unoccupied sites were quenched via incubation with ethanolamine. sPSGL1/Fc-coated biosensors were incubated with various concentrations of sSiglec-5/C4BP (0–20 μg/ml) in octet-buffer (20 mM Hepes, 50 mM NaCl, 2.5 mM CaCl_2_, pH7.0) and association was monitored for 10 min. Subsequently, sensors were transferred to wells containing octet-buffer for 10 min in order to allow dissociation of the complex. In another set of experiments, immobilized sPSGL1 was pre-incubated with sialidase (0.01 U/ml) before incubation with sSiglec-5/C4BP or P-selectin (both 10 μg/ml). In complementary binding experiments, sSiglec-5/C4BP (10 μg/ml) was immobilized onto amine-coupling biosensors and incubated with sPSGL1/Fc, sL-selectin/Fc, sCD44/Fc (10 μg/ml). A non-sialylated bacterial protein was used as negative control.

### *In vitro* perfusion assays

*In vitro* perfusions were performed using a camera-equipped Venaflux-Microfluidic platform, allowing real-time video-monitoring. Perfusions were performed using THP1 cells (which express both PSGL1 and Siglec-5^10^,^48^) or primary leukocytes. Perfusions were performed using the VenaFlux-perfusion system (Cellix Ltd, Dublin, Ireland) or an in-house developed perfusion system^49^. Biochips were coated with E-selectin, P-selectin or VCAM1 (5 μg/ml) and exposed to PSGL1-expressing cells, which were perfused at a shear of 0.5 dyn/cm^2^ or 2 dyn/cm^2^. Where indicated, perfusions were performed in the presence of competing proteins (10–50 μg/ml). Perfusions over BSA- or polyvinylpyrrolidone blocked biochips were used as a control, revealing that rolling and adhesion was less than 5% compared to E-selectin, P-selectin or VCAM1-coated biochips, respectively. Three images of different fields taken at approximately 100s after initiation of the perfusion were used for analysis in all cases.

### Basal leukocyte rolling *in vivo*

Female wild-type C57Bl/6 mice (aged 3–4 weeks) received a single intravenous injection of sSiglec-5/C4BP (0.8 mg/kg) or vehicle in the tail vein. Infusion of sSiglec-5/C4BP had no effect on blood counts, as assessed in 12 mice given vehicle (n = 6) or sSiglec-5/C4BP (n = 6). Leukocyte-counts were 2.8 ± 0.8 × 10^3^ and 2.8 ± 0.5 × 10^3^ cells/μl, respectively. Also red blood counts (7.5 ± 0.4 × 10^6^ and 7.0 ± 1.1 × 10^6^ cells/μl) and platelet counts (920 ± 290 × 10^3^ and 833 ± 189 × 10^3^ platelets/μl) were similar for both groups. The mesenteric vessels of ketamine/xylazine-anesthetized mice were exteriorized (a procedure that takes routinely about 15 min) and exposed venules (25–35 micron) were recorded via video-microscopy for 10 min. Per mouse, 2–3 vessels were monitored. Video-images were then analyzed in a blinded fashion for the number of rolling leukocytes and for the number of adhering leukocytes (defined as adhering for >30 sec).

### Preparation of TNFα-containing gel

Agarose (0.4 g) was diluted in 10 ml of distilled water and heated in a microwave until agarose dissolution was observed (routinely: 1.5 min at 800 W). A volume of 10 ml 2xPBS was warmed and mixed with the agarose-solution to reach a final agarose concentration of 2%. Recombinant murine TNFα (Peprotech, Neuilly-sur-Seine, France) was added to 90 μl of the agarose-solution to a final concentration of 0.32 nM. The gel was kept at 4 °C until use.

### *In vivo* model for TNFα-induced inflammation

Ketamine/xylazin/atropine anesthetized male wild-type C57Bl/6 mice (aged 5 weeks) were cannulated in the jugular vein, and the trachea was intubated to facilitate spontaneous respiration by the mouse. After the scrotum was incised, the testicle and surrounding cremaster muscle were exteriorized. Routinely, this procedure lasts about 20 min. Mice were given the fluorescent dye Syto59 to label leukocytes, and subsequently sSiglec-5/C4BP was applied (0.8 mg/kg) via a single injection through a cannula placed in the jugular vein. Syto59 was used for the staining of neutrophils for two reasons. First, to facilitate the quantitative analysis of leukocyte recruitment and extravasation of 4 vessel segments *per* mouse in parallel. Second, by analyzing the fluorescent signal it is possible to distinguish between leukocytes present in the blood vessel and extravasated leukocytes. By using brightfield illumination, it would be difficult to monitor leukocyte movements in the extravascular space over a 5-h period.

Ten minutes after injection of the protein, a 1-mm^3^ piece of TNFα-containing 2% agarose gel was deposited on a preselected avascular area of the cremaster. Microvessels were observed using an Olympus BX61 WI microscope with a 40× NA water immersion objective. Digital images were captured using a Cooke Sensicam CCD camera in 640 × 480-pixel format using an intravital optical system with spinning disk confocal microscopy. All experiments were performed using at least 3 mice, and at least 4 venules of 20–40 micron were selected for analysis in each mouse. The fluorescence intensity, corresponding to the leukocyte recruitment in the vessel or in the tissue, was determined with the software Slidebook 5.5 (Intelligent Imaging Innovations, Göttingen, Germany).

### Statistics

Single comparisons were performed using two-tailed unpaired t-test or the Mann-Whitney test. Multiple comparisons were performed using 1-way ANOVA followed by Dunnett’s test or Tukey’s test. Differences were considered significant at *p*-values below 0.05.

## Additional Information

**How to cite this article**: Pepin, M. *et al*. Soluble Siglec-5 associates to PSGL-1 and displays anti-inflammatory activity. *Sci. Rep.* **6**, 37953; doi: 10.1038/srep37953 (2016).

**Publisher's note:** Springer Nature remains neutral with regard to jurisdictional claims in published maps and institutional affiliations.

## Supplementary Material

Supplementary Information
