# Supplementary material for: Soluble Siglec-5 associates to PSGL-1 and displays anti-inflammatory activity
Source: Sci Rep. 2016 Nov 28;6:37953. doi: 10.1038/srep37953 (PMC5125011; doi:10.1038/srep37953)
Supplement: Supplementary Information [file srep37953-s1.pdf]

Supplementary information

Soluble Siglec-5 associates to PSGL-1 and displays anti-inflammatory activity

Marion Pepin[1], Soraya Mezouar[2], Julie Pegon[1], Vincent Muczynski[1], Frédéric Adam[1], Elsa P. Bianchini[1], Amine Bazaa[1], Valerie Proulle[1,3], Alain Rupin[4], Jerome Paysant[5], Laurence Panicot-Dubois[2], Olivier D. Christophe[1], Christophe Dubois[2], Peter J. Lenting[1], Cécile V. Denis[1]

[1]Institut National de la Santé et de la Recherche Médicale, UMR_S 1176, Univ. Paris-Sud, Université Paris-Saclay, 94276 Le Kremlin-Bicêtre, France

[2]Aix Marseille Université, Inserm UMR_S 1076, (VRCM) Vascular Research Center of Marseille, 13385 Marseille, France

[3]Department of Biological Hematology, CHU Bicetre, Hopitaux Universitaires Paris Sud, AP-HP, Paris, France

[4]Institut de Recherche International Servier, Recherche Translationelle et Clinique Oncologie, 92150, Suresnes, France

[5]Institut de Recherches Servier, Unité de Recherche et de Découverte Cardiovasculaire, 92150, Suresnes, France

Correspondence

Dr. P.J. Lenting

Inserm U1176, 80 rue du General Leclerc, 94270 Le Kremlin-Bicetre, France

Tel: +331 49 59 56 51; Fax: +331 46 71 94 72

Email: peter.lenting@inserm.fr

Supplementary Figure 1:

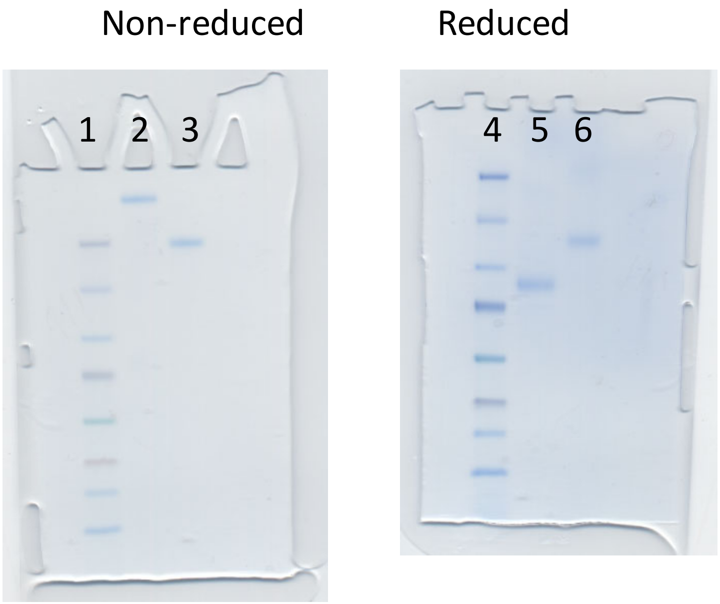

Legend to Fig. S1: Analysis of purified sSiglec-5/C4BP and sSiglec-5/Fc via SDS-Page and Coomassie-staining under non-reduced and reduced conditions. Images represent whole gels before cropping and conversion into black & white images (as how they are presented in Fig. 1). Lanes 1 & 4: molecular weight markers; lanes 2 & 5: sSiglec-5/C4BP; lanes 3 & 6: sSiglec-5/Fc.
